# R-R interval and modification of cardiac output following cardiac surgery: the importance of heart rate optimisation by external pace maker

**DOI:** 10.1186/2197-425X-3-S1-A592

**Published:** 2015-10-01

**Authors:** G Tavazzi, N Bergsland, F Mojoli, M Pozzi, D Gibson, F Guarracino, S Price

**Affiliations:** PhD in Experimental Medicine University, Pavia, Italy; Anesthesia and Intensive Care Division I, Emergency Dpt-Fondazione IRCCS Policlinico San Matteo, Pavia, Italy; IRCCS 'S.Maria Nascente', Don Gnocchi Foundation, Milan, Italy; Royal Brompton and Harefield NHS Foundation Trust, Cardiology Unit, London, United Kingdom; Azienda Ospedaliero-Universitaria Pisana, Det of Anaesthesia and Critical Care Medicine, Pisa, Italy; Royal Brompton and Harefield NHS Foundation Trust, Adult Intensive Care, London, Italy

## Introduction

Inadequate cardiac output is associated with poor outcome following cardiac surgery and is generally modified by use of inotropic agents, volume resuscitation and epicardial pacing. Although temporary epicardial pacing is frequently utilised post-cardiac surgery, the potential impact of determining the optimal R-R interval in the critically ill has largely been ignored. Echocardiography (Echo) has been used to optimise cardiac output by modifying pacing settings.

## Objectives

We sought to determine the impact of the R-R interval on cardiac electromechanics, cardiac output and stroke volume in patients following cardiac surgery on a cardiothoracic intensive care unit.

## Methods

24 sequential patients (14 males, age 65.9 ± 16.5; APACHE 12.6 ± 2.9)being paced following cardiac surgery were assessed using transthoracic echocardiography within 4 hours of returning to the CICU. Pacing was set to the presumed optimal rate basing on clinical evaluation by the treating anesthesiologist. Echo data included LV/RV systolic & diastolic function including Doppler assessment of stroke volume (SV), Cardiac Output (CO) and total isovolumic contraction/relaxation time derived by ejection time (ET) & filling time (FT). tIVT was calculated as [60-(total ET+total FT); t-IVT > 14 s/m is associated with left ventricular dyssynchrony [1]. Echo was performed at the baseline RR interval and at heart rates from 70 to 110 bpm in increments of 10bpm. Time Pearson correlation coefficients, accounting for the repeated nature of the data, were used to assess relationships between the investigated measures.

## Results

Results are shown in Table 1.

**Table 1:** Table 1

Surgery	RR vs CI	LV t-IVT vs CI	LV t-IVT vs CO	LV t-FT vs HR	LV t-ET vs HR
Aortic 11 pts	p 0.065 r -0.28	p < .0001 r -0.69	p < .0001 r -0.83	p <0.001 r 0.72	p <0.001 r 0.72
Mitral 4 pts	p 0.006 r -0.63	p < .0001 r -0.60	p < .0001 r -0.86	p <0.46 r -0.18	p <0.001 r 0.83
CABG 6 pts	p 0.47 r -0.16	p < .0001 r -0.77	p < .0001 r -0.91	p < .021 r -0.5	p <0.09 r 0.37
Right 3 pts	p 0.81 r -0.07	p < .0001 r -0.92	p < .0001 r -0.77	p <0.001 r -0.86	p <0.005 r 0.77

**Figure 1:**
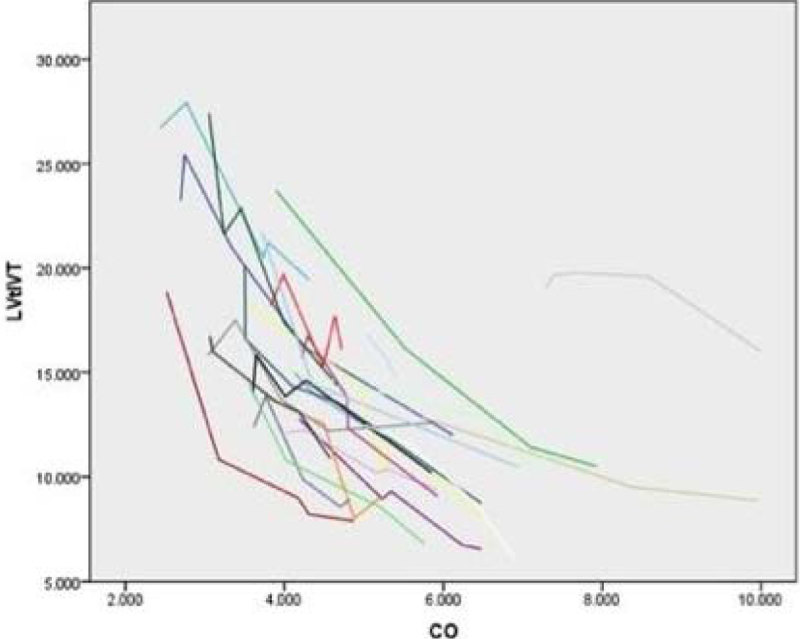
**Lv t-IVT vs CO.**

There was no consistent nor predictable correlation between changes in HR and SV, CO or CI in the overall population studied except in mitral valve surgery patients. A strong linear negative correlation was found between LV t-IVT and SV, CO and CI in all the groups (p < 0.0001 r^2^-0.88 - Figure 1) regardless the Ejection Fraction (EF). LV t-IVT < 14 s/m corresponded always with the best CO.The mean changes in CO and CI overall the echo-driven Pacemaker Optimization were respectively 2.21 ( ± 0.97) and 1.2 ( ± 0.52); the maximum change was in patient 10, CO increase from 3.89 L/min to 7.93 L/min (CI 2.18 to 4.45 L/min /m^2^).

## Conclusions

This is the first report of the role of HR optimization in post-cardiac surgery patients with epicardial wire, which led to changes in pacemaker baseline setting in 79% of the patients. This underlines that the clinical assessment of the pacemaker setting is not sufficient and that the HR has to be set on the patient's features. The t-IVT showed to be a sensitive index to assess the best hemodynamic and electromechanic profile in our population.
